# Nonthermal biocompatible plasma in stimulating osteogenic differentiation by targeting p38/ FOXO1 and PI3K/AKT pathways in hBMSCs

**DOI:** 10.1186/s13036-024-00419-2

**Published:** 2024-05-28

**Authors:** Khadija Akter, Youngsun Kim, Eun Ha Choi, Ihn Han

**Affiliations:** 1 Department of Plasma Bio Display, Plasma Bioscience Research Center, Kwangwoon University, Seoul, 01897 Korea; 2 Department of Electrical and Biological Physics, Plasma Bioscience Research Center, Kwangwoon University, Seoul, 01897 Korea; 3 Department of Obstetrics and Gynecology, Kyung Hee University Medical Center, Seoul, 02447 Korea

**Keywords:** Human bone marrow mesenchymal stem cells, Nonthermal biocompatible plasma, Osteogenic differentiation, PI3K/AKT signaling pathway

## Abstract

Osteoporosis is manifested by decreased bone density and deterioration of bone architecture, increasing the risk of bone fractures Human bone marrow mesenchymal stem cells (hBMSCs)-based tissue engineering serves as a crucial technique for regenerating lost bone and preventing osteoporosis. Non-thermal biocompatible plasma (NBP) is a potential new therapeutic approach employed in several biomedical applications, including regenerative medicine. NBP affects bone remodeling; however, its role in the regulation of osteogenic differentiation in hBMSCs remains largely unexplored. This study aimed to explore the efficiency of NBP in promoting osteogenic differentiation, and the molecular pathways through which these responses occurred in hBMSCs. We found that NBP facilitated osteogenic differentiation through the upregulation of the bone morphogenic protein signal (BMPs) cascade, which in turn induced the expression of p38 and inhibited the forkhead box protein O1 (FOXO1). To further gain insight into the mechanism through which NBP extensively triggers the initiation of osteogenic differentiation in hBMSCs, PI3K/AKT pathway was also analyzed. Overall, these results highlight that NBP enhances osteogenic differentiation in hBMSCs by the stimulation of the p38/FOXO1 through PI3K/AKT signaling pathways. Therefore, the application of NBP in hBMSCs may offer tremendous therapeutic prospects in the treatment of bone regeneration and osteoporosis prevention.

**Supplementary Information:**

The online version contains supplementary material available at 10.1186/s13036-024-00419-2.

## Introduction

Postmenopausal osteoporosis (PMOP) is a bone metabolism condition related to estrogen deficiency, primarily affecting postmenopausal women, and is characterized by altered skeletal microarchitecture and poor bone structure [1, 2]. According to age-standardized prevalence statistics, the osteoporosis hazard of elderly men and women in Chinese urban was 2.68% and 13.82%, respectively [3]. Osteoporotic fractures are a major cause of impairment, and death and are major public health concerns [4, 5]. In addition, bone diseases and congenital malformations are becoming increasingly common and continue to be of concern for musculoskeletal and orthodontic specialists. Despite autogenous bone grafting, the recommended surgical treatment for severe periodontal disease has undergone substantial improvement [6]. However, conventional knee replacement medications are plagued by several intractable drawbacks, such as surgical stress, lack of donors, donor-site illness, and infections. [7, 8]. Bacterial extracellular vesicles (BEVs) containing therapeutic compounds have surfaced as a hopeful substitute in bone tumor therapy and bone related diseases. Nonetheless, the lack of a well-defined standard for producing, isolating, and characterizing BEVs is still a topic of controversy [9]. To overcome these restrictions, bone tissue regeneration approaches have provided novel therapeutic alternatives, including tissue formation, scaffold-free cell sheet applications, and cytokine activation for considerable bone abnormalities and diseases treatment [10–12]. Mesenchymal stem cells (MSCs), key components of osteogenesis with the potential for multilineage differentiation and adaptability, including bone and cartilage are crucial for the repair of skeletal abnormalities [13, 14]. Mesenchymal stem cells from bone marrow, appear to have low contributor incidence and can harvest easily, making them potential candidates for healing major bone cracks among the plentiful sources [15–17]. Importantly, hBMSCs are utilized to treat a range of ailments, including bone deformities, osteoarthritis, and arthritic, while also possessing the potential to alter bone homeostasis [18–20]. However, the osteogenic potential of hBMSCs in bone healing remains unclear. Therefore, a greater comprehension of the mechanism behind hBMSC bone formation may assist in establishing a novel approach for preventing and controlling osteoporosis. Recent research reports suggested that human stem cells are effective through the reconstruction of cartilage and bone regeneration when used in combination with nonthermal plasma treatment [21].

A partly ionized substance comprising biomolecules, electrons, ions, as well as exciting molecules (along with neutral particles and radicals) is referred to as a non-thermal biocompatible plasma (NBP) [22, 23]. NBP brought in a significant deal of research to the domain of tissue regeneration considering the possibility that reactive species released during plasma treatment may positively affect cell growth and differentiation. NBP has been suggested as a potential tool for stimulating bone regeneration [24, 25], accelerating wound healing [26–28], stimulating immune responses, and apoptosis in cancer cells [29–31]. The therapeutic value of plasma techniques for regeneration medicine is greatly determined by the reactor conception, gaseous composition, plasma power, along additional factors associated with treatment. Reactive oxygen species, also known as ROS, and reactive nitrogen species, or RNS, in particular, are produced by NBP, and they might trigger multiple proteins associated with signaling networks to regulate the functions of cells. It is conceivable that the generation of reactive species released through various plasma discharge systems [32–34], considerably improves and controls metabolic activity, multiplication, and differentiation [35, 36]. However, to date, no research has investigated the mechanism through which NBP contributes the osteogenic differentiation in hBMSCs. Prior research has employed osteoprogenitor cells to hypothesize the osteogenic differentiation capacity of cells exposed to atmospheric plasma, characterized by their biocompatibility and bioactivity. [36]. Moreover, we elucidated that direct exposure of cells to NBP not only instigates osteogenic differentiation in human bone marrow mesenchymal stem cells (hBMSCs), but also enhances osteogenesis efficiency by modulating the surface properties of scaffolds when biomaterials are subjected to NBP treatment [37]. Based on these findings, our objective was to elucidate the mechanistic underpinnings through which NBP orchestrates osteogenic differentiation in hBMSCs, while concurrently assessing its prospective efficacy as a pharmacotherapeutic candidate. Consequently, our present inquiry provides evidence suggesting that NBP induces stem cell differentiation under disparate conditions, distinct from those governing precursor cells, thereby amplifying its regulatory role in orchestrating osteogenic differentiation within hBMSCs. To test our hypothesis, we compared the efficiency of NBP alone or in combination with mineral supplements through in vitro screening, including assessment of the expression of osteogenic marker genes/proteins, calcium (Ca) mineral deposition, and immunofluorescence staining. We further screened and identified the influence of NBP on cellular responses and the involvement of molecular signaling pathways during osteogenic differentiation. NBP demonstrated outstanding consequences in enhancing the osteogenic functions of hBMSCs by stimulating bone morphogenic proteins (BMPs), p38/forkhead box protein O1 (FOXO1) and PI3K/AKT signaling pathways in vitro while maintaining stem cell characteristics. We predict these results support the conceptual groundwork to explore potential use of NBP regarding hBMSCs-based regenerative medicine.

## Results

### Plasma device parameters

**Fig. 1:**
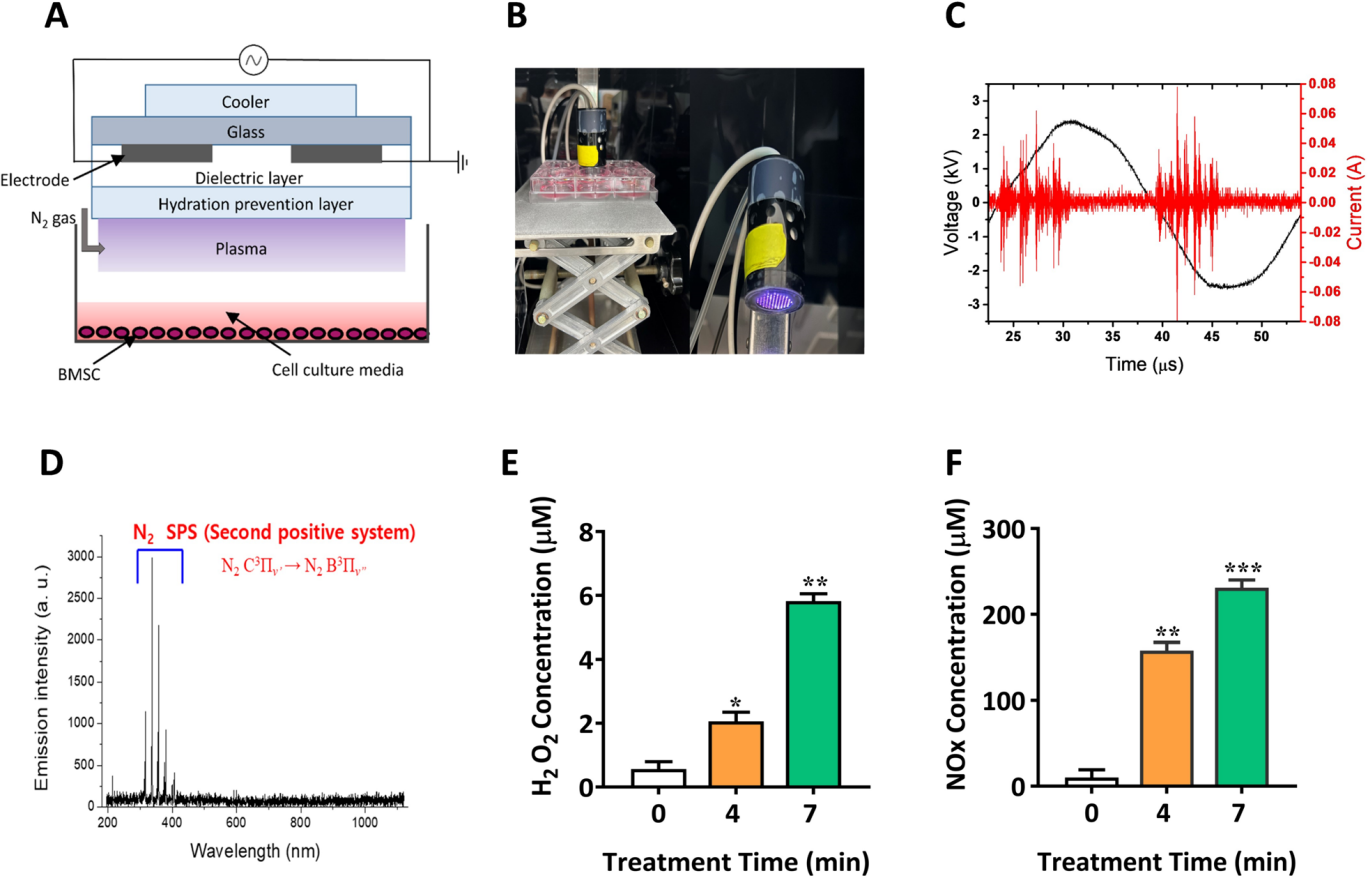
Schematic structure and dielectric properties of the experiment. **A** Graphical representation of a micro DBD source. **B** and **C** Voltage-current waveform, and **D** optical emission spectrum (OES) assessment of DBD using N_2_ gas. The generation of reactive species **E** H_2_O_2_ and **F** NOx in the culture medium of hBMSCs after 4 and 7 min of plasma treatment. The mean and standard deviation values are used to represent the experimental results. The statistical significance of the data points was assessed using the student’s t-test, and significant differences were indicated by *p < 0.05; **p < 0.01, and ***p < 0.001 vs the untreated control

Figure 1A shows a diagrammatic illustration of the dielectric barrier discharge (DBD) source. Figure 1C showed the voltage and current waveforms generated by DBD. Figure 1D illustrates optical emission spectroscopy using the nonthermal plasma, which depicts the intensity of the emission lines at different wavelengths and can be used to figure out the species involved throughout the plasma discharge. To estimate RONS generation in a liquid cell culture medium driven by DBD plasma, we assessed H_2_O_2_ and NOx concentrations (Fig. 1E, F). The amount of H_2_O_2_ in the medium was measured in response to DBD plasma after 4 and 7 min of treatment. The result showed that the H_2_O_2_ concentration in culture media with 4 min treatment induced 1.8 μM (*p* < 0.05), and 7 min treatment induced 5.8 μM (*p* < 0.01), which is highly significant. Moreover, the NO radical concentration increased to 180 μM (*p* < 0.01) in the 4 and 7 min of DBD treatments yielded a significant value of 250 μM (Fig. 1F, *p* < 0.001) in comparison with the untreated reference.

### Intracellular ROS and RNS contents in hBMSCs

**Fig. 2:**
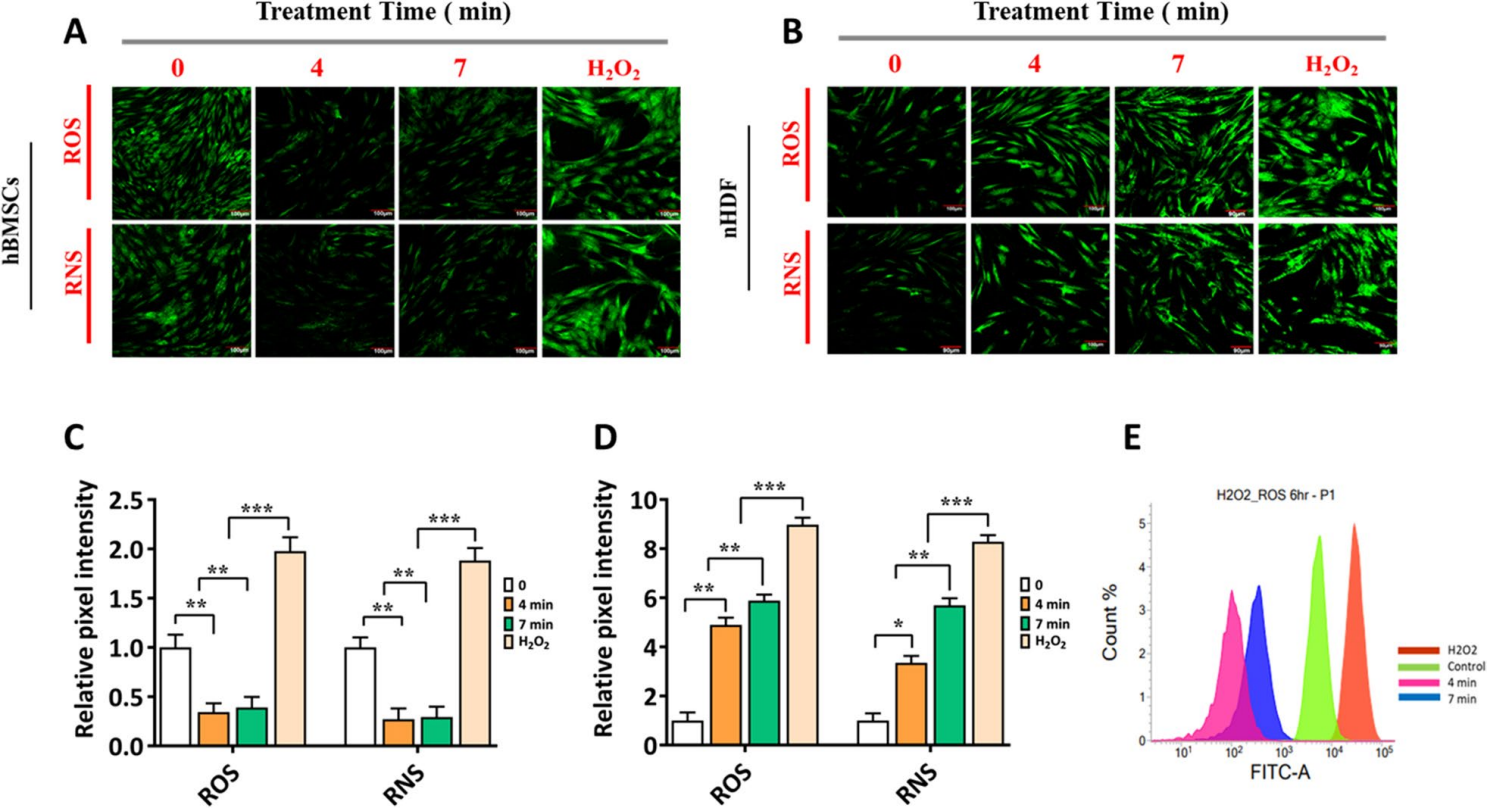
Fluorescence images of intracellular ROS and RNS generation in **A** hBMSCs and **B** nHDF cell lines after 6 h of NBP treatment at 4 and 7 min, respectively, untreated as control, H_2_O_2_ (100 µM) as the positive control. ROS and RNS were detected in cells using H_2_DCFDA and DAF-FM assay kits (scale bars: 90 μm). The confocal pixel intensity was quantified using the ImageJ software as follows: **C** hBMSCs and **D** nHDF cell lines. The hBMSCs were cultivated without an osteogenic inductive medium and data were presented as fold-change normalized to the pixel intensity level of the control. **E** Flow cytometer histogram H_2_DCFDA-stained hBMSCs. The relative intensity of intracellular ROS concentration in hBMSCs was obtained by flow cytometer after 6 h of NBP treatment for 4 and 7 min, untreated as control, with H_2_O_2_ (100 µM) as the positive control. The mean and standard deviation values are used to represent the experimental results. The statistical significance of the data points was assessed using the student’s t-test, and significant differences were indicated by *p < 0.05; **p < 0.01, and ***p < 0.001 vs the untreated control

The formation of intracellular RONS was investigated to determine the optimal conditions for our research. To test the hypothesis that RONS play a role in controlling cellular activity and osteogenic differentiation, we initially measured the production of intracellular RONS in hBMSCs cells after 6 h of NBP treatment. Cellular H_2_DCF-DA and DAF-FM assays were used to detect intracellular ROS and RNS generation in hBMSCs and nDHF after NBP treatment for 4 or 7 min (Fig. 2A, B). Interestingly, NBP treatment effectively reduced the formation of RONS in hBMSCs, which was consistent with the enhanced osteogenic differentiation of hBMSCs. Compared to the untreated control, NBP decreased H_2_DCFDA fluorescence intensity (indicating the quantity of ROS) by 1.4- to 2-fold (*p* < 0.001) in hBMSCs (Fig. 2C). In the normal cell group, NBP plasma treatment considerably increased the levels of intracellular RONS by 2- and 3-fold (*p* < 0.001; Fig. 2D). Further, significantly higher intracellular RONS levels were detected in NBP-treated normal cells than in stem cells. We further examined the production of ROS in hBMSCs using flow cytometry, as described for the NBP treatment. The flow cytometry results demonstrated a shift in the histograms plotting the frequency distribution of H_2_DCFDA fluorescence intensities against the total number of events or cells, which represented a reduction in ROS levels in hBMSCs (Fig. 2E). In this assay, the generation of intracellular ROS decreased with increasing plasma treatment. Moreover, the ROS concentration was lower in the cells treated for 4 and 7 min than in the untreated control.

### Influence of NBP on cell viability, apoptosis and osteogenic differentiation in hBMSCs

**Fig. 3:**
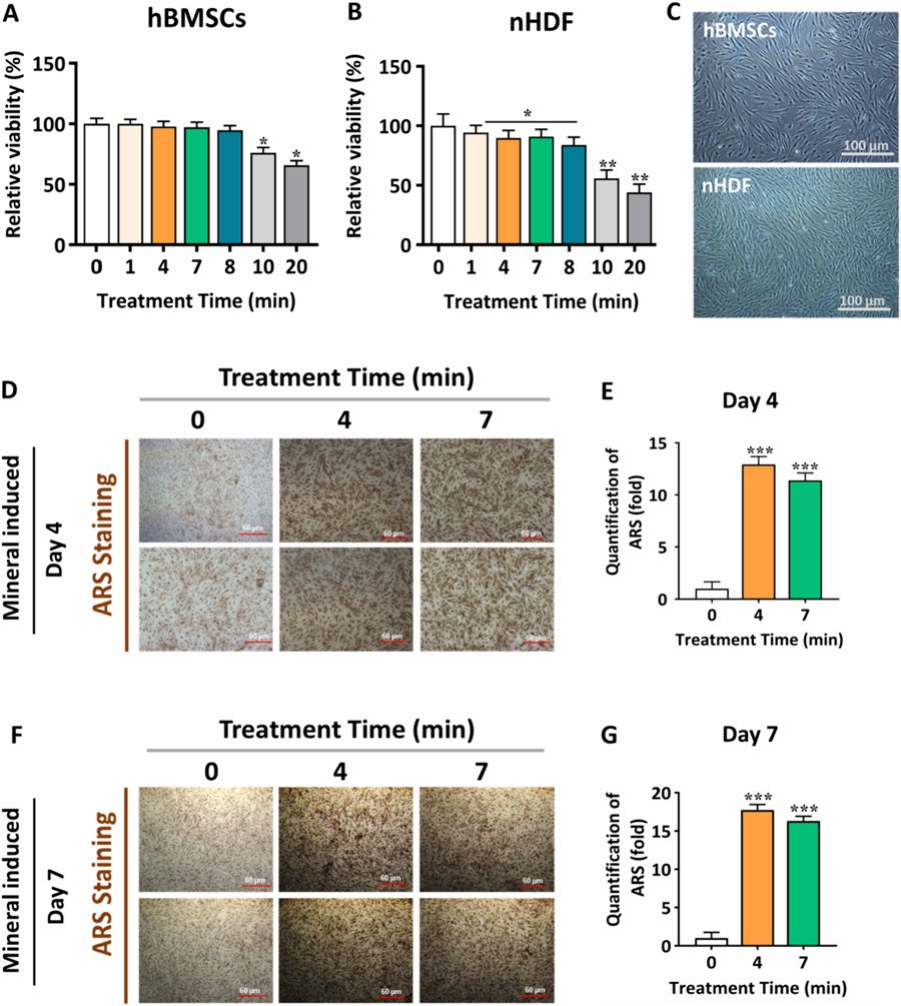
The influence of NBP on osteogenic differentiation of hBMSCs. The cytotoxicity of **A** hBMSCs and **B** nHDF cell lines after NBP exposure times of 1, 4, 7, 8, 10, and 20 min, respectively. **C** Representative images of hBMSCs and nHDF cell morphology were observed under the light microscope. **D** and **F** Representative images of Ca deposition in hBMSCs via Alizarin Red Staining at days 4 and 7 under the light microscope. Scale bars, 100 µm. **E** and **G** Quantified areas of ARS staining measured by ImageJ software. The mean and standard deviation values are used to represent the experimental results. The statistical significance of the data points was assessed using the student’s t-test, and significant differences were indicated by *p < 0.05; **p < 0.01, and ***p < 0.001 vs the untreated control

The cytotoxicity of NBP on hBMSCs and nHDF cells was assessed using the alamarBlue assay. The viability of the hBMSCs remained stable for up to 8 min after 24 h of NBP treatment (Fig. 3A). in addition, 10 and 20 min of plasma treatment result showed in reduction of cell viability by more than 75 and 60%, respectively (*p* < 0.05). In contrast to the results using nHDF, NBP treatment exhibited minimal effects on cell viability for up to 8 min; however, the differences were not significant (Fig. 3B). Interestingly, both cell lines responded to DBD plasma treatment with nearly the same sensitivity. In addition, cell morphology was observed; typically, cells attained more than 80% confluence on days 3–4. Under a microscope, we found that the nHDFs grew exponentially and developed a filopodia-shaped, fibroblast-like appearance (Fig. 3C). hBMSCs grew slowly and demonstrated a spindle-shaped morphology 4 d after harvest in Fig. 3C. Furthermore, annexin V/PI staining was used to analyze apoptosis rate in response to NBP treatment in hBMSCs. To determine the apoptotic rate cells were collected after 24h of NBP treatment. The findings demonstrated that there were no obvious differences was observed in cell population among the NBP treatment groups and the control group. The percentage of viable cells rate was 99.07% and 99.14%, respectively in the 4 and 7 min treatment group (Fig. S1). However, the group exposed to 10 min treatment exhibited a slight reduction in the percentage of viable cells (98.66%) compared to the other treatment groups (Fig. S1). Therefore, we applied 4 and 7 min NBP treatment in the following investigations.

To test the accelerating effect of NBP on hBMSCs, ARS staining was performed as a marker of osteogenic differentiation to detect mineralization levels. The hBMSCs started to develop mineralized Ca nodules after 4 and 7 d of osteogenic differentiation (indicated by the red color in Fig. 3D, F. The cells were harvested with a chemical cocktail consisting of dexamethasone, β-glycerol phosphate and ascorbic acid, and treated as a combination of NBP. Interestingly, when hBMSCs were subjected to a combination treatment, they significantly contributed to the formation of extracellular matrix mineralization on 4 and 7 d of induction compared to the control group (Fig. 3D, F). The mineralization level was quantified by measuring the Ca areas using ImageJ software. Ca secretion was significantly upregulated at 4 and 7 min in the treatment group and attenuated much stronger staining intensity (Fig. 3E, G).

### NBP treatment stimulates p38 and FOXO-1 signaling pathways in hBMSCs

**Fig. 4:**
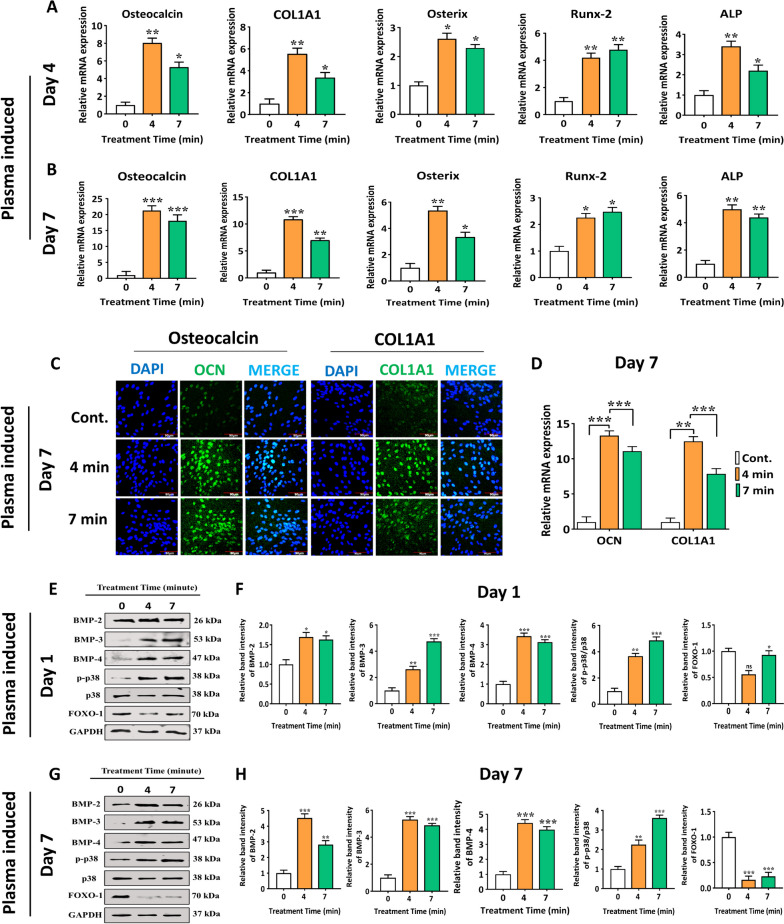
NBP treatment promotes the osteogenic differentiation in hBMSCs. **A**, **B** The messenger RNA (mRNA) levels of osteogenic genes, including OCN, COL1A1, OSX, Runx-2, and ALP, were measured using RT-qPCR. Total RNA was extracted from hBMSCs at two-time points: 4 and 7 d following combination treatment of NBP at 4 and 7min treatment time and mineral supplement (0.08 g/ml of ascorbic acid, 0.30611 g/ml of β-glycerol phosphate, 0.051 M dexamethasone). GAPDH was used as the internal control. **C**, **D** Immunofluorescence staining was performed to detect OCN and COL1A1 protein expression with the administration of a combination treatment of NBP and mineral supplements. OCN and COL1A1 (green) elevated on day 7 of osteogenic differentiation in response to the combination treatment of NBP at 4 and 7 min with a mineral supplement. The green fluorescence represents the OCN and COL1A1, and DAPI is represented by the blue fluorescence. ImageJ software was used to quantify the fluorescence intensities of OCN and COL1A1. The scale bars used in the imaging were 60 µm. The hBMSCs were cultivated for 1 and 7 d after NBP treatment at 4 and 7min with mineral supplements. **E**-**H** the protein expression of BMP-2, BMP-3, BMP-4, p-p38, and FOXO1 was analyzed via western blotting. The quantification of band intensities is represented in the graphs. GAPDH was used for the normalization. The mean and standard deviation values are used to represent the experimental results. The statistical significance of the data points was assessed using the student’s t-test, and significant differences were indicated by *p < 0.05; **p < 0.01, and ***p < 0.001 vs the untreated control

The hBMSCs were cultured for 4 and 7 d, and messenger RNA (mRNA) expression was analyzed to assess the efficiency of NBP treatment. The results of qRT-PCR demonstrated a remarkable upregulation of osteogenic markers OCN (*p* < 0.001, *p* < 0.01), COL1A1 (*p* < 0.01, *p* < 0.5), OSX (*p* < 0.01), RUNX2 (*p* < 0.01), and ALP (*p* < 0.01, *p* < 0.5) on day 4 and 7, with a higher upregulation was observed on day 7 (Fig. 4A and B). Most of these genes exhibited the same pattern of upregulated expression during osteogenic differentiation. To detect the potential genes involved in the NBP-modulated osteogenic differentiation under mineral supplements, additional gene expression analysis was carried out. The results confirmed that NBP, supplemented with minerals, successfully induced hBMSCs into the osteogenic lineage (Fig. S2).

Further immunofluorescence studies were conducted to corroborate the expression of OCN and COL1A1 proteins. The findings demonstrated that NBP treatment increased protein levels of OCN (*p* < 0.001, *p* < 0.001) and COL1A1 (*p* < 0.01, *p* < 0.001)) on day 7 at 4 and 7min (Fig. 4C). The fluorescence intensities of OCN and COL1A1 were measured using ImageJ software (Fig. 4D, F). To further verify the influence of the combination treatment of NBP and a mineral supplement, an immunofluorescence assay was conducted on OCN and COL1A1 protein expression in Fig. S3. The outcomes imply that there was a synergistic effect on osteogenic differentiation in hBMSCs when NBP and mineral supplements were combined. The study also delved into the analysis of protein levels of BMP-2, BMP-3, and BMP-4 as well as the phosphorylation of p-38 and FOXO1 to further investigate the underlying mechanisms. However, hBMSCs were cultivated without mineral supplements after NBP treatment (4 and 7 min) for 1 and 7 d. Without mineral supplementation, NBP treatment significantly altered the levels of BMP-2 (*p* < 0.001), BMP-3 (*p* < 0.001), BMP-4 (*p* < 0.001) and phosphorylated p-38 (*p* < 0.01) in the cells, whereas the levels of FOXO1 (*p* < 0.001) substantially decreased (Fig. 4E-H). The combination of NBP and mineral supplements was found in stimulating BMPs- and p-38-mediated osteogenic differentiation of hBMSCs (Fig. S4). Next, we analyzed the time-dependent effects of NBP on phosphorylated p-38 and revealed a significant upregulation without mineral supplementation in the cells (Fig. S5). These results imply that NBP, with or without mineral supplements, may participate in stimulating BMPs- and p-38-mediated osteogenic differentiation of hBMSCs.

### SB203580 reversed the enhancing effect of NBP on the osteogenic differentiation of hBMSCs through inhibiting the p38 pathway

**Fig. 5:**
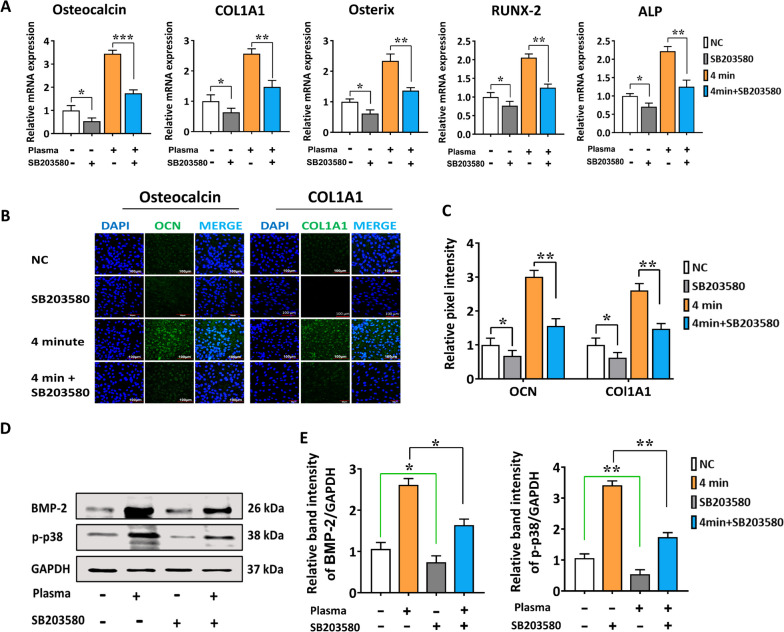
Inhibition of p38 pathways reversed the enhancing effects of NBP on osteogenic differentiation of hBMSCs. **A** The relative mRNA expression of OCN, COL1A1, OSX, Runx-2, and ALP in hBMSCs, they were cultivated in culture media for 2 d with or without SB203580 (10 μm) or NBP (4 min) and quantified via qRT-PCR. GAPDH was used as the internal control. **B** Immunofluorescence staining of OCN and COL1A1 and, **C** its relative pixel intensities on day 2, scale bars: 100 μm. **D** Results of western blot analysis of phosphorylated p38, and BMP-2. **E** Represents the quantification of band intensity on day 2 were performed. GAPDH was used for the normalization. The mean and standard deviation values are used to represent the experimental results. The statistical significance of the data points was assessed using the student’s t-test, and significant differences were indicated by *p < 0.05; **p < 0.01 compared with NC, compared with NBP (4 min)

We used an inhibitor of p-38 (SB203580,10 µM) to explore the inhibitory effects on osteogenic differentiation to confirm the significance of p38. hBMSCs grown in normal culture media for 2 d with or without NBP treatment (4 min) or SB203580 to measure and compare the osteogenic differentiation ability. The addition of SB203580 reversed the mRNA levels of osteogenic markers: OCN, COL1A1, OSX, RUNX-2 and ALP, which were induced through NBP treatment (Fig. 5A). Furthermore, immunofluorescence results supported the observation as shown in Fig. 5B, C. Similarly, western blot findings demonstrated SB203580 reversed the enhancing effects of NBP treatment (4 min) on phosphorylated p-38 and BMP-2 protein levels compared with NC group (Fig. 5D-F). Therefore, the positive effects of NBP on hBMSCs osteogenic differentiation were reversed by blocking p38MAPK pathway.

### NBP stimulated the PI3K/AKT signaling pathways to accelerate the osteogenic differentiation of hBMSCs

**Fig. 6:**
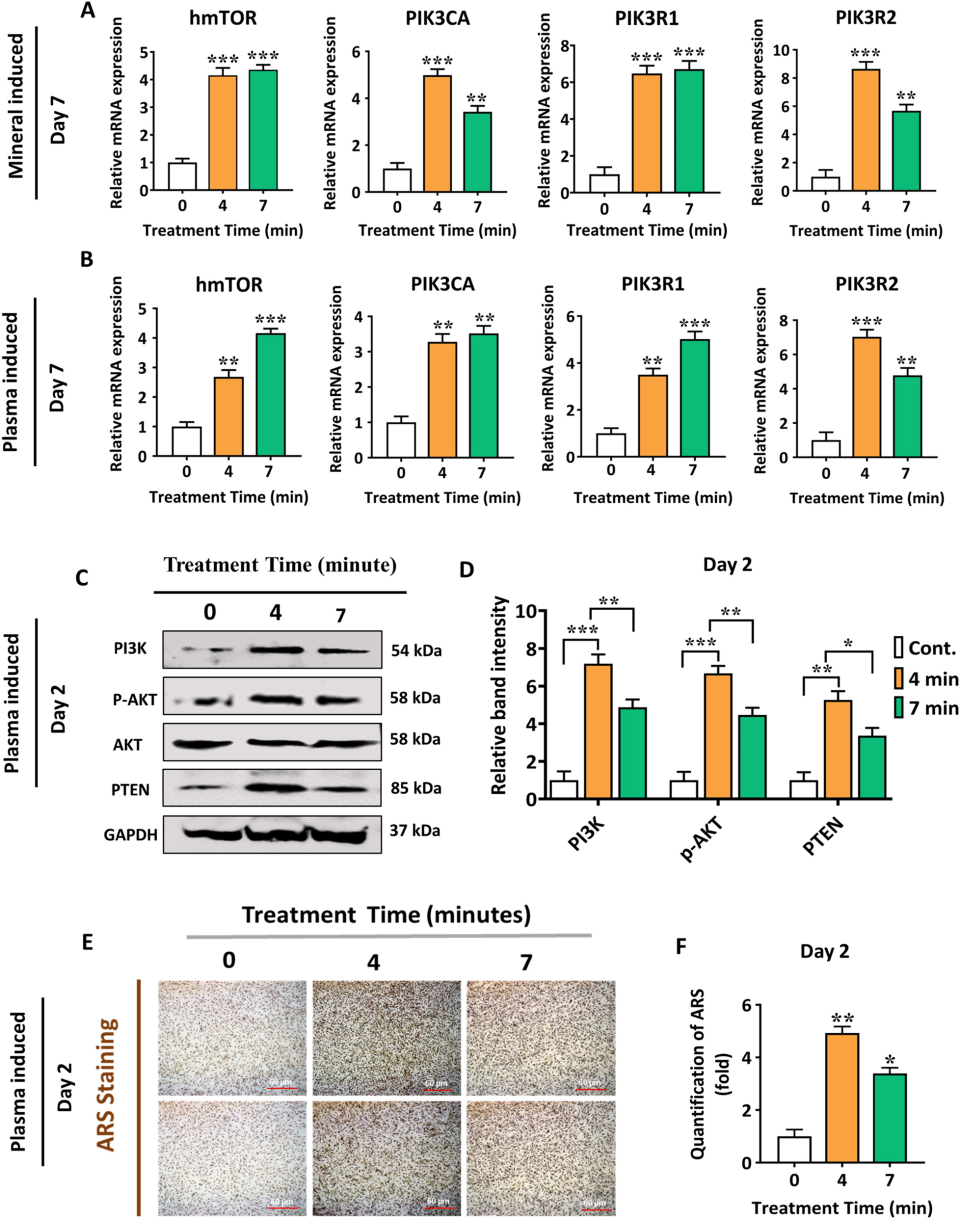
NBP treatment promoted hBMSCs osteogenic differentiation through the activation of PI3K/AKT pathways. **A**, **B** The relative mRNA expression of hmTOR, PIK3CA, PIK3R1 and PIK3R2 is upregulated in hBMSCs quantified via qRT-PCR. Total RNA was extracted from hBMSCs at two-different points: combination treatment of NBP at 4 and 7min treatment time and mineral supplement (0.08 g/ml of ascorbic acid, 0.30611 g/ml of β-glycerol phosphate, 0.051 M dexamethasone) and without mineral supplements following NBP at 4 and 7min treatment time. GAPDH was used as the internal control. **C**, **D** Western blot analysis was performed to detect PI3K, p-AKT, and PTEN protein expression with the administration of NBP treatment at 4 and 7min on day 2. **D** The quantification of band intensities is represented in the graphs. GAPDH was used for the normalization. **E**, **F** Representative images of Ca deposition in hBMSCs via Alizarin Red Staining at day 2 under the light microscope. Scale bars, 60 µm. **F** Quantified areas of ARS staining measured by ImageJ software. The mean and standard deviation values are used to represent the experimental results. The statistical significance of the data points was assessed using the student’s t-test, and significant differences were indicated by *p < 0.05; **p < 0.01, and ***p < 0.001 vs the untreated control

The cells were analyzed for the expression of hmTOR, PIK3CA, PIK3R1, and PIK3R2 via qRT-PCR to learn more about the function of NBP in tissue regeneration. hBMSCs were cultured in the mineral supplements and complete medium containing 4 and 7 min NBP treatment for 7 d. The combination treatment contained mineral supplements including 0.08 g/ml of ascorbic acid, 0.30611 g/ml of β-glycerol phosphate, 0.051 M dexamethasone with NBP treatment at 4 and 7 min. The mRNA expression levels differed at each time point. However, a higher osteogenic differential potential was demonstrated by NBP combination treatment when subjected to mineral supplementation compared to NBP treatment alone, as indicated by the upregulated mRNA expression (Fig. 6A, B).

To further gain a deeper understanding of the mechanism, how NBP alone, without mineral supplementation, significantly contributes to the initiation of osteogenic differentiation in hBMSCs, we analyzed the protein levels of PI3K, p-AKT, and PTEN via western blot analysis. However, hBMSCs were cultured in the complete medium without mineral supplements after NBP treatment (4 and 7 min) for 2 d. In (Fig. 6C, D), NBP significantly enhanced the phosphorylation of AKT, PI3K, and PTEN with 4 and 7 min treatment time. In addition, mineralization assay confirmed that hBMSCs triggered by NBP treatment as well as developed more mineralization compared to the untreated control group (Fig. 6E, F). All of these findings suggested that NBP activated the PI3K/AKT pathways and promoted the osteogenic differentiation of hBMSCs.

### The efficiency of NBP on hBMSCs osteogenic differentiation are hindered by PI3K/AKT inhibition

**Fig. 7:**
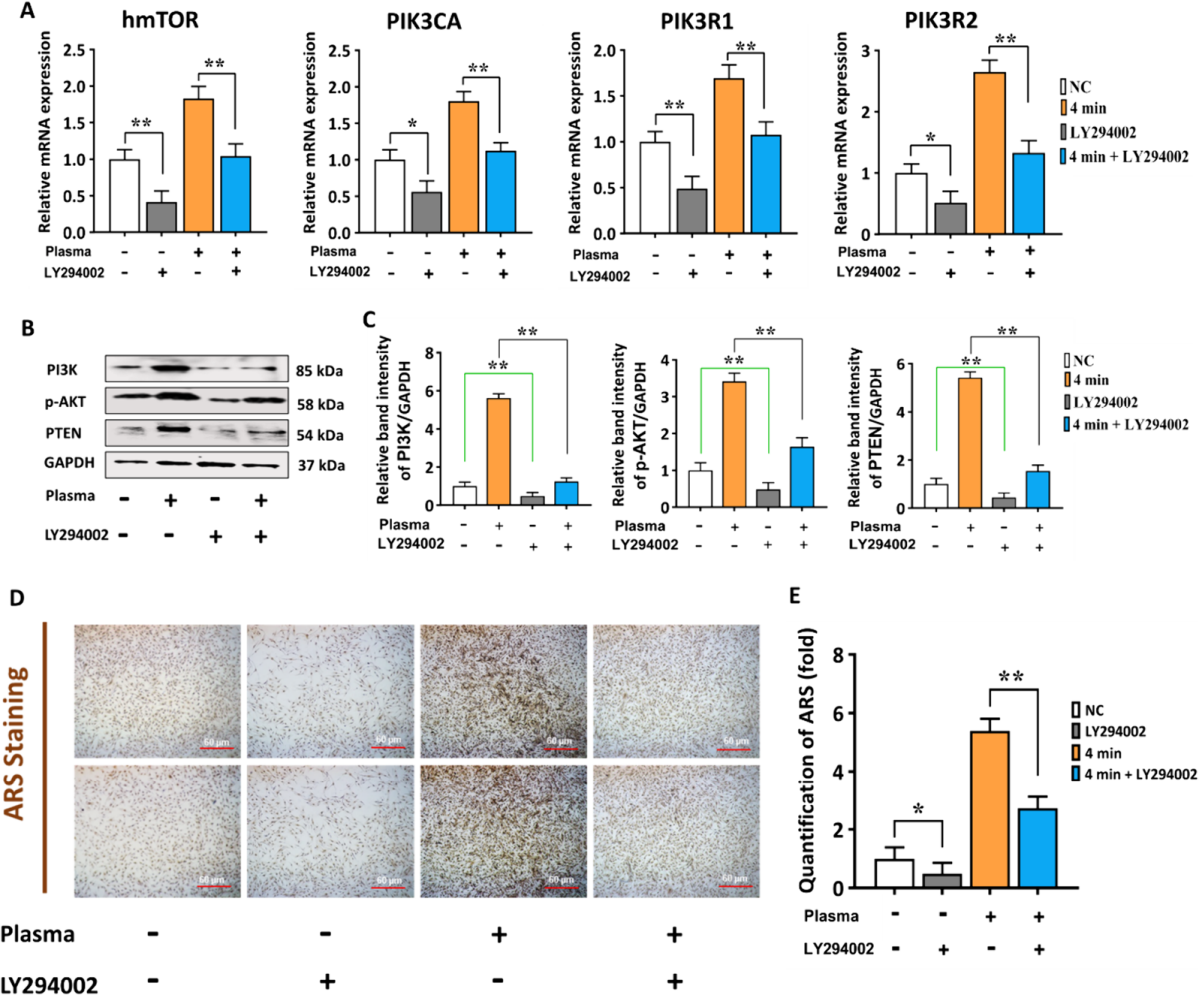
LY294002 reversed the stimulating effect of NBP and blocked the PI3K/AKT pathways. **A** The amount of gene levels of hmTOR, PIK3CA, PIKR1, and PIK3R2 in hBMSCs, were cultivated in culture media for 2 d with or without LY294002 (10 μm) or NBP (4 min). hBMSCs were cultured with or without NBP treatment at 4 min or LY294002 (10 μm); **B** Results of western blot analysis of PI3K, phosphorylated AKT, and PTEN. **C** Represents the quantification of band intensity on day 2 were performed. GAPDH was used for the normalization. **D** Representative images of Ca deposition in hBMSCs via Alizarin Red Staining on day 2 under the light microscope. Scale bars, 100 µm. **E** Quantified areas of ARS staining measured by ImageJ software. The mean and standard deviation values are used to represent the experimental results. The statistical significance of the data points was assessed using the student’s t-test, and significant differences were indicated by *p < 0.05; **p < 0.01 compared with NC, compared with NBP (4 min)

In the following experiment, we added LY294002 (PI3K/AKT) inhibitor to the medium to block with the effects of NBP on PI3K/AKT pathways. The hBMSCs were cultured in the normal culture media for 2 d with or without NBP treatment (4 min) or LY294002 to measure and compare the osteogenic differentiation ability. The addition of LY294002 rescued the mRNA levels of hmTOR, PIK3CA, PIK3R1, and PIK3R2 which were stimulated through NBP treatment (Fig. 7A). The results of western blot demonstrated that LY294002 reversed the stimulating effects on PI3K, p-AKT and PTEN (Fig. 7B, C). Moreover, mineralization assay was suppressed by the blocking of PI3K/AKT signaling in hBMSCs (Fig. 7D, E). Therefore, following NBP treatment, the PI3K/AKT pathways were found to be significant in stimulating osteogenic differentiation of hBMSCs, as demonstrated by the outcomes presented above.

### PI3K/AKT signaling pathways regulates p38-induced osteogenic differentiation

**Fig. 8:**
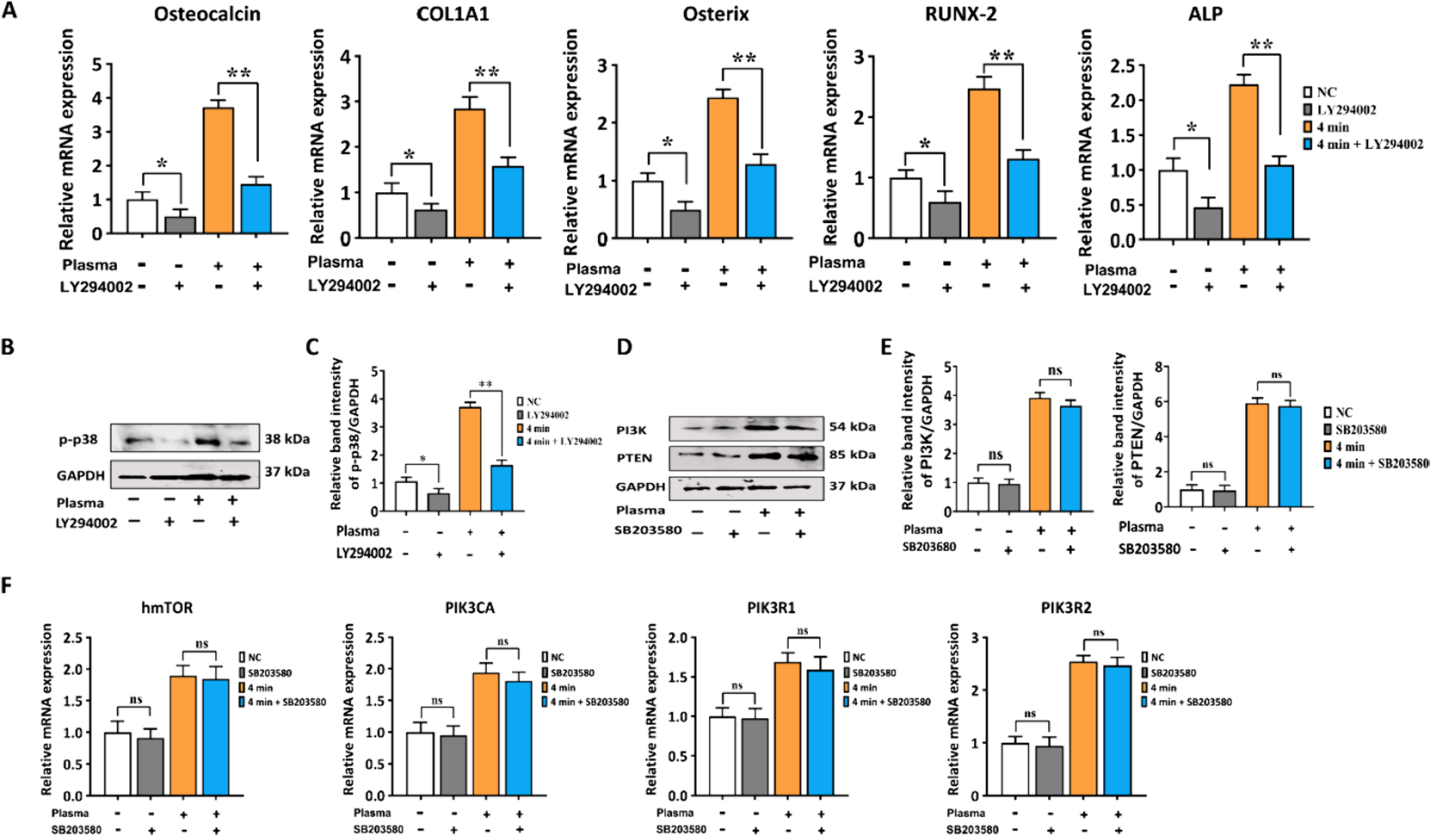
The comparison of inhibitory effects on p38 and PI3K/AKT signal translocation by the PI3K/AKT and p38 inhibitors. **A** The relative mRNA expression of OCN, COL1A1, OSX, Runx-2, and ALP in hBMSCs was measured by qRT-PCR. Cells were cultured in complete media for 2 d with or without LY294002 (10 μm) or NBP (4 min). **B** Results of western blot analysis of phosphorylated p-38. **C** Representing the quantification of band intensity on day 2 were performed. GAPDH was used for the normalization. **D**–**F** hBMSCs were cultured with or without NBP treatment at 4 min or SB205380 (10 μm); **D** Results of western blot analysis of PI3K and PTEN. **E** Represents the quantification of band intensity on day 2 were performed. GAPDH was used for the normalization. **F** The relative mRNA expression of hmTOR, PIK3CA, PIKR1, and PIK3R2 in hBMSCs, were cultivated in culture media for 2 d with or without SB205380 (10 μm) or NBP (4 min). The mean and standard deviation values are used to represent the experimental results. The statistical significance of the data points was assessed using the student’s t-test, and significant differences were indicated by *p < 0.05; **p < 0.01 compared with NC, compared with NBP (4 min)

To further explore the potential role of PI3K/AKT in the effectiveness of NBP treatment, we blocked the p38 pathway by using the PI3K/AKT (LY294002) inhibitor. The protein level of p38 is a critical member of MAPK pathway was analyzed, to explore whether NBP stimulated osteogenic differentiation is regulated by the PI3K/AKT pathways. We treated the cells with NBP and its control group (NC) with LY294002 inhibitor for 2 d to analyze and compare the osteogenic differentiation capability. Blocking the PI3K/AKT signaling pathway inhibited the NBP induced osteogenic markers (OCN, COL1A1, OSX, Runx-2 and ALP) (Fig. 8A). Western blot analysis demonstrated that treatment of cells with PI3K/AKT inhibitor (LY294002) altered the amount of phosphorylated p38 (Fig. 8B, C). In addition, to further verify the relationship between NBP and p38 as well as PI3K/AKT pathways, we added the p38 inhibitor (SB203580) in hBMSCs. However, the results from the western blot and qRT-PCR, we found that SB203580 did not produce similar inhibitory effects in the protein levels of PI3K and PTEN as the LY294002 (PI3K/AKT) inhibitor (Fig. 8E, F). Therefore, the above results confirmed that NBP attenuated osteogenic differentiation of hBMSC is regulated through PI3K pathways.

## Discussion

Osteogenesis is an essential component of bone remodeling and plays a role in various metabolic changes and therapeutic circumstances [38]. It has been demonstrated that NBP holds promise as an innovative treatment approach for diverse tissue regeneration goals. Through its influence on cell growth and specialization, NBP can stimulate differentiation of stem cells by affecting various cellular processes, making it an intriguing candidate for tissue engineering [39]. This may include modulation of signaling pathways, gene expression, and the release of specific bioactive molecules. The specific mechanisms by which NBP affects stem cell differentiation can vary depending on the type of stem cells involved and the experimental conditions. NBP has been proven effectively in promoting the differentiation of various stem cell types, such as mesenchymal stem cells, adipose tissue-derived stem cells, pluripotent stem cells [40–42]. In addition, it serves as a potent tool for promoting the proliferation of various adult stem cells derived from mesoderm while preserving their stemness and pluripotency, adding value to stem cell therapy. Furthermore, NBP can induce the differentiation of progenitor cells into different cell types, including neurons [43], periodontal ligaments [44], and osteoblasts [36]. These findings align with and support our results, can provide a border perspective on the overall efficacy of NBP’s impact on the stem cell differentiation. The multifaceted effects of NBP across different cell types and tissues emphasize its potential as a comprehensive approach in the field of regenerative medicine.

Our study supports the proposed application of NBP to be a highly effective medicine for the prevention of osteoporosis-related disorder. Particularly, NBP releases a great deal of ROS and RNS, which are essential for the formation of progenitor cells. Therefore, it is essential to examine the manner in which NBP promotes the osteogenesis of MSCs. However, this investigation is the first to take into the consideration the use of NBP, which produces various reactive species that considerably affect the stimulation osteogenesis of hBMSCs by the p38 and PI3K/AKT pathways. This study used a novel µ-DBD generated plasma technology to treat the hBMSCs and cultured them with and without mineral supplements. The potential cytotoxic effects of NBP were evaluated before analyzing its effects on osteogenic differentiation in hBMSCs. hBMSCs were not cytotoxic to the (0–10) min treatment time, although osteogenic differentiation could efficiently induce (Fig. 3). Bone remodeling requires mineralization. In vitro, initiation of bone tissue formation (osteogenesis) generates extracellular Ca deposits. These deposits were verified as successful in vitro bone formation by ARS staining [45]. Subsequently, the amount of Ca deposition was measured using mineralization assay, which serves as a marker of the late stage of osteogenesis and can reflect the degree of osteogenic differentiation. We found NBP markedly accelerated the formation of extracellular matrix mineralization and attenuated much stronger staining intensity on days 4 and 7 when hBMSCs were subjected to a combination treatment (Fig. 3). Normal cells continuously produce low levels of ROS. ROS production is significantly upregulated in response to various physiological conditions. Excessive ROS generation can trigger the oxidation of molecular interactions and cellular dysfunction, which ultimately causes cell death [46]. Assessment of ROS is extremely difficult owing to their short lifetimes. We examined the production of intracellular RONS and found that ROS generation is related to cell differentiation and homeostasis. In addition, oxidative stress was assessed in our study using data from confocal microscopy and flow cytometry (Fig. 2A-E).

Runx-2 is crucial for osteogenic differentiation and osteoblast function that controls the expression of OCN, COL1A1, and OSX, whereas ALP serves as an initial indicator during the early phase of this process [47, 48]. To further explore the osteogenic ability of hBMSCs with the treatment of NBP alone or in combination of mineral supplement, we conducted qRT-PCR to explore potential gene regulating the therapeutic effect of NBP on hBMSCs. The mRNA findings indicated a significant increase in the levels of genes associated to the process of osteogenesis after treatment of hBMSCs with combination of mineral supplements or in NBP alone for 4 and 7 d (Fig. 4, Fig. S2). The findings imply that treatment of NBP with mineral supplements is a highly effective and efficient agent that stimulates both the early and late phases of hBMSCs differentiation. To determine the extent to which NBP without any mineral supplements contributes to triggering osteogenic differentiation in hBMSCs, we measured osteogenic genes and proteins expression on days 4 and 7 in culture medium. The mRNA expression levels increased at each time point; however, the effects were less pronounced in the group treated solely with NBP than in the group that received the combination treatment (Fig. 4). The BMP signaling pathway has emerged as a new hotspot and revolution in the prevention of osteoporosis as a fundamental and crucial mechanism for controlling the osteogenic differentiation of hBMSCs. BMP is a transforming growth factor (TGF) superfamily member and can accelerate cell growth and gene regulation, thus aiding mesenchymal cells differentiate into osteoblasts [49, 50].

To understand the mechanism underlying the influence of NBP treatment on osteogenesis under combination of mineral supplement or in NBP alone, we investigated the protein levels of BMP2/BMP3/BMP4 cascade and phosphorylated p38 and FOXO1 in hBMSCs on day1 and 7 (Fig. 4, Fig. S4). We noticed that the indicated protein expression levels were strongly upregulated on day 7, and this upregulation was time-dependent. These outcomes bring us to come to the conclusion that NBP alone or with mineral supplements could potentially expedite the process of skeletal development and facilitate the osteogenesis of hBMSCs by actively participating in all phases from initiation to maturation. We blocked the osteogenic gene and phosphorylated p38 protein with the related inhibitor SB203580 and observed the corresponding effects on hBMSCs, to further confirm the contribution of NBP-mediated activation of p38 route towards the osteogenic differentiation. On the basis of gene expression, SB203580 was found to reverse the osteogenic markers OCN, COL1A1, OSX, Runx-2 and ALP, which were stimulated through NBP treatment. Moreover, SB203580 rescued the enhancing effects of NBP (4 min) on phosphorylation of p38 compared with negative control (NC) (Fig. 5). To advance more effective medicines for the treatment of skeletal abnormalities in humans, it is crucial to understand the molecular pathways that control bone homeostasis. There is evidence from numerous research, PI3K/AKT pathway is crucial for controlling the bone development [51]. However, no research has explored how PI3K/AKT pathway and NBP interact to promote bone regeneration. Interestingly, we found NBP treatment alone or in the combination of NBP and mineral supplementation showed a stronger osteogenic differential potential, as seen by the increased mRNA expression (Fig. 6). Therefore, we further conducted ARS staining to figure out the role of NBP alone without any mineral supplement in enhancing hBMSCs osteogenic differentiation.

To gain additional insight into the mechanism by which NBP encourages osteogenic differentiation, we added LY294002, an inhibitor of PI3K/AKT to illustrate the influence of NBP to osteogenic differentiation of hBMSCs. LY294002 may partially mitigate promoting effects of NBP (Fig. 7). To further confirm NBP-induced osteogenic differentiation is controlled by PI3K/AKT pathways, we treated the cells with NBP and its control group (NC) with LY294002 and SB203580, p38 inhibitor. The LY294002 inhibitor block osteogenic markers and p38-MAPK pathway, as indicated by gene expression and western blot evidence, however SB203580 inhibitor did not show any inhibitory effects on PI3K/AKT pathways (Fig. 8).

**Scheme 1:**
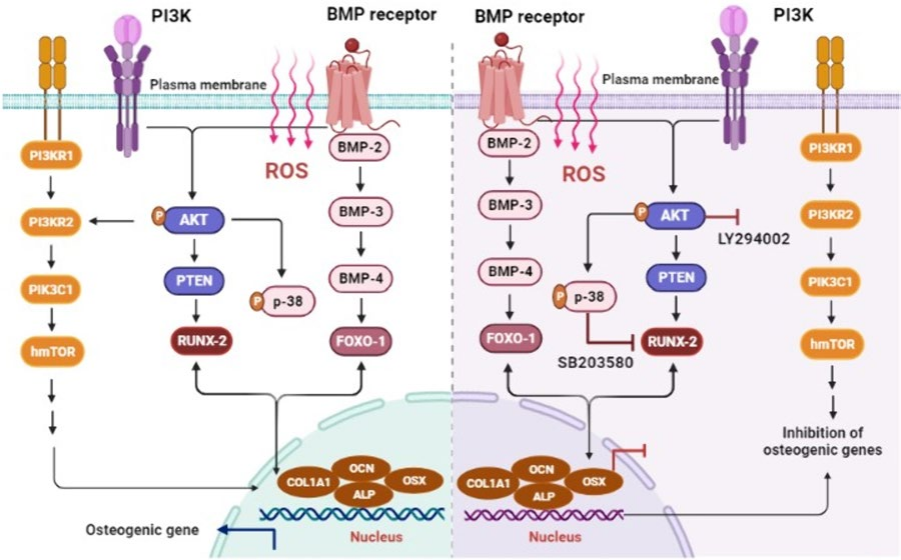
Graphic illustration of a possible mechanism for the manner in which NBP induced osteogenic differentiation in hBMSCs. It was discovered that NBP causes the production of ROS, which drives PI3K/AKT signaling pathways and stimulates osteogenic genes and proteins linked to osteogenic differentiation

In summary, NBP is considered a pioneering source for therapeutic tissue regeneration, offering several advantages that make it appealing for the treatment of complex bone diseases. Investigating the potential positive influence of reactive species released during plasma treatment on hBMSCs, which possesses properties suitable for bone regeneration. Our findings demonstrated that NBP effectively heightened the induction of an osteogenic phenotype, including increased the mRNA levels of osteogenic gene (OCN, COL1A1, Osterix, Runx-2 and ALP) and the expression of key osteogenic markers in hBMSCs (Fig. 4). In this context, we established the significance of the p-38 /FOXO-1 and PI3K-AKT signaling pathway in NBP-induced osteogenic differentiation of hBMSCs, providing novel evidence supporting the use of hBMSCs in bone tissue engineering (Scheme 1). Our research shed light on the molecular mechanisms responsible for the stimulated osteogenic differentiation of hBMSCs and suggested its potential role in inhibiting osteoporosis (OP). Despite a great deal of interest in NBP research, the therapeutic uses of NBP (particularly MSCs-derived from bone marrow) in the treatment of osteoporosis still require more investigation. However, on the basis of our findings, NBP may laid the groundwork to bring a novel therapeutic option in the treatment of complex bone diseases and provide an effective paradigm in the filled of biomedical research. In this study, we only conducted in vitro screening to explore the influence of NBP on hBMSCs. The research did not extend to animal models, which are essential to enhance the practical relevance and effectiveness of NBP treatment. Therefore, future research will involve mice models to thoroughly explore the fundamental mechanisms behind the enhancement of bone formation by NBP, with the aim of assessing its potential therapeutic application in osteoporosis diseases.

## Conclusion

In tissue engineering, hBMSCs are a key source of osteogenic progenitor cells and have promising potential for applications in the regeneration and repair of bone grafts. The results of this study provide evidence that NBP efficiently enhances the osteogenic differentiation of hBMSCs, either by alone or in combination with mineral supplements. Specifically, NBP treatment stimulated extracellular matrix mineralization, promoted a high level of tissue mass, and upregulated the gene expression and the protein contents related to osteogenic markers. Mechanistically, we discovered that NBP treatment reinforced hBMSCs osteogenic differentiation through stimulation of PI3K and AKT pathway. Therefore, the application of NBP could potentially render hBMSCs-based bone tissue repair to prevent bone loss and osteoporosis.

## Materials and methods

### Plasma device and treatment

A typical micro-dielectric barrier discharge (μ-DBD) of a plasma source was utilized in this research, which includes electrodes with a thickness of 5 μm, electrode gap distance of 200 μm, and aluminum oxide or Al_2_O_3_ layer of 1 μm thickness to prevent electrode hydration during discharge. Figure 1A shows the micro dielectric barrier discharge (DBD) configuration and other experimental details. An alternating-current power supply was used to generate the NBP with a discharge voltage of 500 V and a discharge current of 13 mA. For NBP generation, nitrogen gas (N_2_) was used as the feed gas at a flow rate of 1.69 L per minute (lpm). A spectrometer (model HR4000, Ocean Optics, Dunedin, FL, USA) was used to measure the optical emission spectrum (OES) of the plasma produced by the DBD (Fig. 1C). The intensity of the light emitted from the device was recorded in terms of wavelength. The diameter of the plasma discharge area was approximately 35 mm. In Fig. 1B, the treatment distance between the discharge surface and the media surface was approximately 2–3 mm.

### Cell culture and maintenance

The American Type Culture Collection (ATCC, Manassas, VA, USA) provided a preliminary culture of hBMSCs and human dermal fibroblasts (nHDF) obtained from Promo cells. The hBMSCs were harvested in complete α- MEM media (HyClone Laboratories, Logan, UT, USA), consisting 15% fetal bovine serum (FBS, Opti-Gold, USA), 1% antibiotic (penicillin/streptomycin, Gibco, USA), and basic fibroblast growth factor (bFGF, Corning, USA). Human dermal fibroblast cells were maintained in fibroblast basal medium (FBM, Lonza, USA) with 10% heat-inactivated fetal bovine serum and 1% antibiotics. All cells were maintained under humidified incubator at 37 °C and 5% carbon-dioxide atmosphere. After reaching 80% confluence, adherent cells were digested with trypsin and passed through another stage. The cells were then expanded for a maximum of ten passages and used in subsequent experiments.

### Osteogenic differentiation and cells treatment

Briefly, to promote osteogenesis, hBMSCs were cultured in mineral supplement. The formulation of osteogenic differentiation content comprised 10% FBS, 0.08 g/ml of ascorbic acid-2-phosphate, 0.30611 g/ml of β-glycerol phosphate, 0.051 M dexamethasone (Sigma-Aldrich, St Louis, MO, USA) supplemented with α-MEM media. hBMSCs were cultured in this medium for 4–7 d to stimulate cortical mineralization. Sigma-Aldrich provided the targeted PI3K/AKT inhibitor LY294002, to inhibit AKT phosphorylation. SB203580, a p-38 inhibitor was purchased from Sigma-Aldrich to block the p-38 phosphorylation. In the 4 min plasma treatment group, SB203580 and LY294002 (10 μM) was added to the culture media with or without inhibitor. The cells were harvested for 2 d and collected for analysis of RNA, protein and osteogenic differentiation.

### Mineralization assay

Alizarin Red S (ARS) staining was performed to visualize Ca deposition in hBMSCs. To identify Ca accumulation in the extracellular matrix, the cells were plated and cultured in NBP treatment for 2 d and combination of osteogenic induction medium for 4 and 7 d. Following fixation, the cells were stained with 40 mM Alizarin red staining solution (Santa Cruz Biotechnology, Dallas, TX, USA) at room temperature. The Ca deposits were imaged using a fluorescence microscope (Nikon, Melville, NY, USA).

### Cell metabolic activity detection (Alamar Blue assay)

Alamar Blue, a redox fluorogenic indicator of metabolic reduction (Invitrogen, Thermo Fisher Scientific), was used to detect cellular metabolic activity. The third passage of hBMSCs and nHDF cells was seeded into a 6-well plate (SPL Life Sciences, Korea) and incubated for 24 h to reach confluence prior to the experiment. The cells were then treated with DBD plasma at designated time points (1, 3, 4, 7, 10, and 20 min). We considered the control group to be untreated. Alamar Blue solution was added with α-MEM media and incubated for 3 h after 24 h plasma treatment. The medium was transferred to 96-well black plates and the optical density was measured using a microplate reader (Biotek, VT, USA) at an excitation wavelength of 540 nm and an emission wavelength of 600 nm.

### Annexin-V/PI staining

To investigate the effects of NBP on hBMSCs cell apoptosis, cells were harvested in 6-well plates at a seeding density of 2 × 10^5^ cells per well. The assessment of hBMSCs apoptosis was performed through Annexin V/propidium iodide (PI) staining (BD Biosciences Pharmingen, CA, USA), following the provided instructions from the manufacturer. The apoptosis rate in hBMSCs was assessed at various time points (0, 4, 7, and 10 min) following 24h of NBP treatment. Briefly, cells were collected by 0.5% EDTA-free trypsin (Hyclone SH30042.01) and washed twice with cold PBS. The cells were then re-suspended in 1 × binding buffer. Afterward, the cells were stained with 5 µl Annexin V-FITC and 5 µl propidium iodide for 15 min in the dark at room temperature, and flow cytometry (BD Biosciences, USA) was performed within 1h.

### Determination of reactive oxygen and nitrogen species (RONS)

ROS production levels were measured using 2′7′-dichlorodihydrofluorescein diacetate (H_2_DCFDA; Invitrogen, USA) in hBMSCs cells. The DAF-FM assay kit (Invitrogen, USA) was used as a total RNS indicator according to the manufacturer’s instructions. The cells were cultured in 6-well plates for cell fluorescence detection after preparation as described above (Corning, Midland, NC, USA). The cells were incubated with 100 M hydrogen peroxide (H_2_O_2_) for 30 min to generate a positive control group. The cells were incubated with 10 μM H_2_DCFDA and DAF-FM probes for 30 min in the dark condition, and fluorescence intensity was observed under a confocal laser scanning microscope (Olympus, Hamburg, Germany). To further confirm our results, we prepared samples at the same procedure and then incubated them for 6 h to detect the intracellular ROS. Subsequently, each group of cells was collected for detection on flow cytometry.

The most substantial effects on stem cell differentiation were caused by H_2_O_2_ and nitric oxide (NO). We measured the extracellular H_2_O_2_ and NO levels in serum-free culture media following NBP treatment using the QuantiChrom TM Peroxide Assay Kit and the Nitric Oxide Assay Kit (Bio Assay System, CA, USA), respectively. Experimental procedures were performed in accordance with the manufacturer’s instructions.

### Ribonucleic acid (RNA) isolation and quantitative reverse-transcriptase Polymerase Chain Reaction (qRT-PCR)

Total cellular ribonucleic acid (RNA) was extracted by following the instruction of RNA iso plus (Takara Bio Inc. Japan) after 4 and 7 d of differentiation, and the quantification of osteogenic gene expression levels in hBMSCs. Samples were prepared with basic α-MEM media and mineral supplements after treating the cells with DBD plasma. Based on the manufacturer’s instructions, 2 μg of an RNA sample was prepared for reverse transcription into cDNA using the Prime Script RT Reagent Kit (Takara Bio Inc.). Quantitative reverse-transcription Polymerase Chain Reaction (qRT-PCR) was used to determine the expression levels of osteogenic genes using iQ SYBR Green Super Mix PCR Mixture (Bio-Rad Laboratories, USA). Glyceraldehyde 3-phosphate dehydrogenase (GAPDH) was used as the internal control. The amplification was operated by following the PCR cycling protocol: 95 °C for 15 s and 60°C for 60 s, followed by 40 cycles. Further, the 2-∆∆Ct technique was used to determine the relative target gene expression levels. The primer sequences for qRT-PCR are listed in Table S1.

### Western blotting analysis

Protein samples were isolated from hBMSCs using radioimmunoprecipitation assay (RIPA) lysis buffer (Tech-Innovation, South Korea). The protein concentration for quantitative analysis was measured using the bicinchoninic acid protein concentration measurement kit. Subsequently, 20 µg cell lysate was separated by 4–15% sodium dodecyl sulfate-polyacrylamide gel electrophoresis (SDS-PAGE) (BIO-RAD), followed by transfer into nitrocellulose membrane (Millipore, MA, USA) and blocked with 5% bovine serum albumin for 2 h at room temperature. Subsequently, the membrane was probed with the following rabbit primary antibodies: BMP-2, BMP-3, BMP-4, FOXO1, p-38, p-p38, PI3K, AKT, p-AKT, PTEN and GAPDH (Cell Signaling Technology, USA) at 4 °C overnight. After washing, the blots were incubated with horseradish peroxidase-conjugated goat anti-rabbit secondary antibodies (Cell Signaling Technology). Protein expression was visualized using an excellent chemiluminescent substrate or ECL kit (Bio-Rad, Italy) and a chemiluminescence detection system (Thermo Scientific, USA). The density of the blots was determined using GAPDH as the control. Protein quantification was performed using the ImageJ software.

### Immunofluorescence

Immunofluorescence was used to observe the expression and distribution of proteins throughout cells. The cells were grown in a 6-well plate at a density of 2 × 10^5^ and OCN and COL1A1 were detected using confocal microscope after combination of osteogenic induction on day 4 and 7 as well as nonthermal plasma treatment on day 7. Briefly, the cells were fixed with 4% formaldehyde at room temperature for 15 min. The cells were then permeabilized with 0.1% 100X Triton and blocked with 5% bovine serum albumin (BSA) for an additional 30 min. The prepared cells were then treated with the primary antibodies OCN (1:500) and Col1A1 (1:500) (Cell Signaling Technology, USA) for a single night at 4°C following three PBS washes. A secondary antibody, Alexa Fluor 488-phalloidin (Abcam, Germany), was used to localize the actin at room temperature for 2 h. Subsequently, the cells were visualized using a confocal laser scanning microscope OFV10-ASW (Olympus, Hamburg, Germany).

### Statistical analysis

The findings were described using the mean and standard deviation. Each experiment was performed at least three times. GraphPad Prism 8.0.2 software was used to perform the data analysis. The statistical significance of the data points was assessed using the student’s t-test, and significant differences were indicated by * *p* < 0.05; ** *p* < 0.01, and *** *p* < 0.001 vs the untreated control.

### Supplementary Information

**Supplementary Material 1.**

**Supplementary Material 2.**

## Data Availability

No datasets were generated or analysed during the current study.
