# Supplementary material for: Nonthermal biocompatible plasma in stimulating osteogenic differentiation by targeting p38/ FOXO1 and PI3K/AKT pathways in hBMSCs
Source: J Biol Eng. 2024 May 28;18:35. doi: 10.1186/s13036-024-00419-2 (PMC11134625; doi:10.1186/s13036-024-00419-2)
Supplement: Supplementary file 1 — Supplementary Material 1. [file 13036_2024_419_MOESM1_ESM.docx]

**Supplementary Information**

Nonthermal biocompatible plasma in stimulating osteogenic differentiation by targeting p38/ FOXO1 and PI3K/AKT pathways in hBMSCs

Khadija Akter^1,2,†^ , Youngsun Kim^3,†^, Eun Ha Choi^1,2,*^ and Ihn Han^1,2,*^

^1^Department of Plasma Bio Display, Kwangwoon University, Seoul 01897, Korea.

^2^Plasma Bioscience Research Center, Kwangwoon University, Seoul 01897, Korea.

^3^Department of Obstetrics and Gynecology, Kyung Hee University Medical Center, Seoul 02447, Korea.

*Corresponding Author(s) Email(s): hanihn@kw.ac.kr (I. H.);

ehchoi@kw.ac.kr (E. H. C.)

Contributing Authors: [santaafrin02@gmail.com](mailto:santaafrin02@gmail.com); chacha0725@naver.com

† These authors contributed equally

| 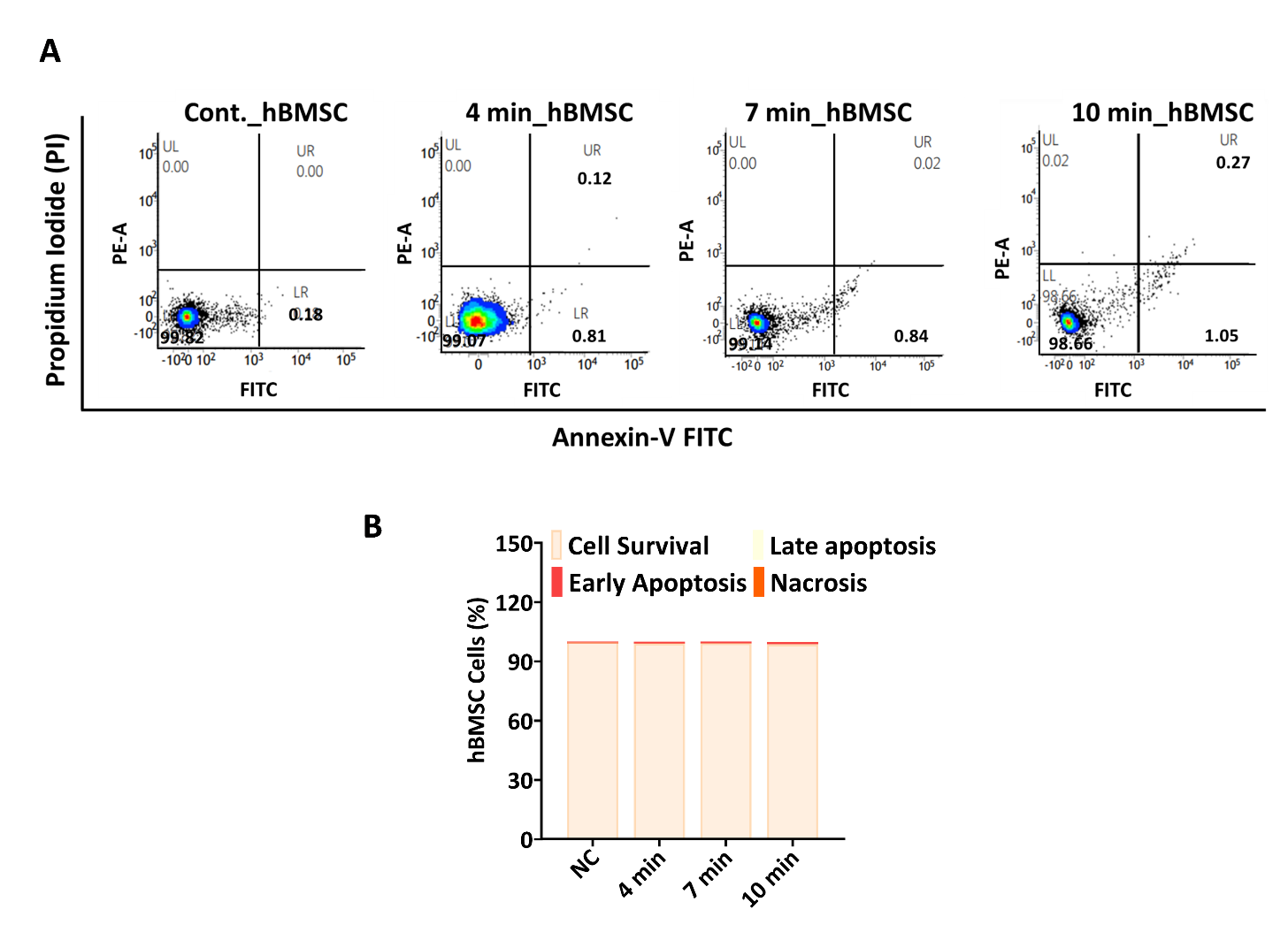 |
| --- |
| **Fig. S1** The possible effects of NBP on the apoptosis of hBMSCs. (A-B) Cell apoptosis in hBMSCs was analyzed using Annexin-V/PI staining. The scatter plots represent PI (Y-axis) and annexin V-FITC (X-axis). The apoptosis rate in hBMSCs was assessed at various time points (0, 4, 7, and 10 minutes) following 24h of NBP treatment. Cells in early apoptosis are distinguished by Annexin V-FITC, which detects externalized phosphatidylserine, while PI is used to identify cells that have compromised plasma membrane integrity, such as necrotic or late apoptotic cells. |

|  |
| --- |
| **Fig. S2** Osteogenic differentiation of hBMSCs is promoted by the combination of mineral supplements with NBP treatment. (A–B) The messenger RNA (mRNA) levels of osteogenic genes, including OCN, COL1A1, OSX, Runx-2, and ALP, were measured using RT-qPCR. Total RNA was extracted from hBMSCs at two time points: 4 and 7 d following combination treatment of NBP at 4 and 7min treatment time and mineral supplement (0.08 g/ml of ascorbic acid, 0.30611 g/ml of β-glycerol phosphate, 0.051 M dexamethasone). GAPDH was used as the internal control. The mean and standard deviation values are used to represent the experimental results. The statistical significance of the data points was assessed using the student's t-test, and significant differences were indicated by * p < 0.05; ** p < 0.01, and *** p < 0.001 vs the untreated control. |

| 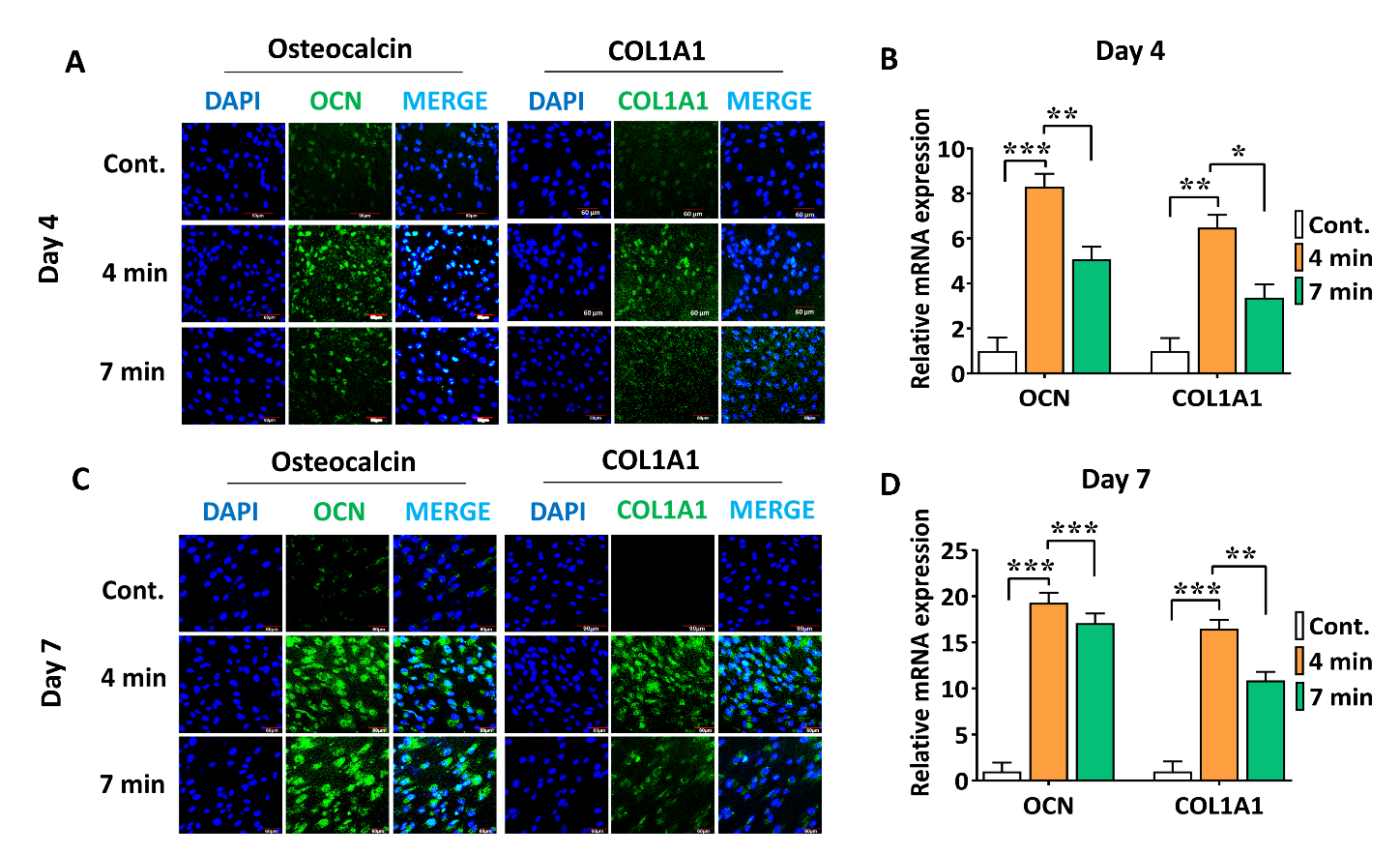 |
| --- |
| **Fig. S3** (A–D) Immunofluorescence staining was performed to detect OCN and COL1A1 protein expression with the administration of a combination treatment of NBP and mineral supplements. The hBMSCs were harvested at two time points: 4 and 7 d following combination treatment of NBP at 4 and 7min treatment time and mineral supplement (0.08 g/ml of ascorbic acid, 0.30611 g/ml of β-glycerol phosphate, 0.051 M dexamethasone OCN and COL1A1 (green) elevated on days 4 and 7 of osteogenic differentiation in response to the combination treatment of NBP at 4 and 7 min with a mineral supplement. The green fluorescence represents the OCN and COL1A1, and DAPI is represented by the blue fluorescence. (B–D) ImageJ software was used to quantify the fluorescence intensities of OCN and COL1A1. The scale bars used in the imaging were 60 µm. The statistical significance of the data points was assessed using the student's t-test, and significant differences were indicated by * p < 0.05; ** p < 0.01, and *** p < 0.001 vs the untreated control. |

| 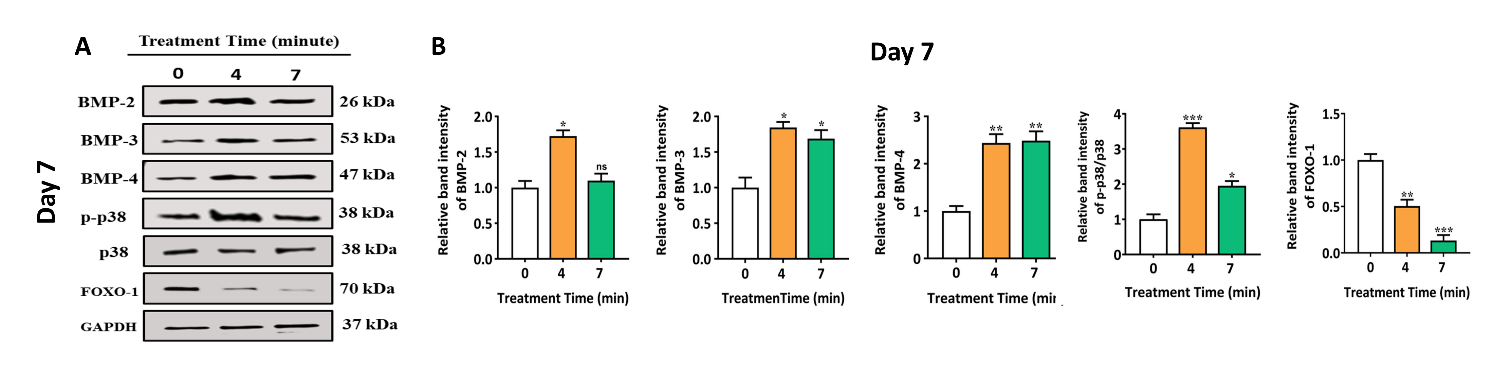 |
| --- |
| **Fig. S4** NBP with mineral treatment promotes osteogenic differentiation in hBMSCs through the stimulation of the BMPs and p-38 signaling pathway. (A) hBMSCs were cultivated for 7 d after NBP treatment at 4 and 7min with mineral supplements and the protein expression of BMP-2, BMP-3, BMP-4, p-p38, and FOXO-1 was analyzed via western blotting. (B) The quantification of band intensities is represented in the graphs. The findings showed that BMP-2, BMP-3, BMP-4, and p-p38 elevated and knockdown FOXO-1 protein levels in the treated group as compared to the control. GAPDH was used for the normalization. The mean and standard deviation values are used to represent the experimental results. The statistical significance of the data points was assessed using the student's t-test, and significant differences were indicated by * p < 0.05; ** p < 0.01, and *** p < 0.001 vs the untreated control. |

| **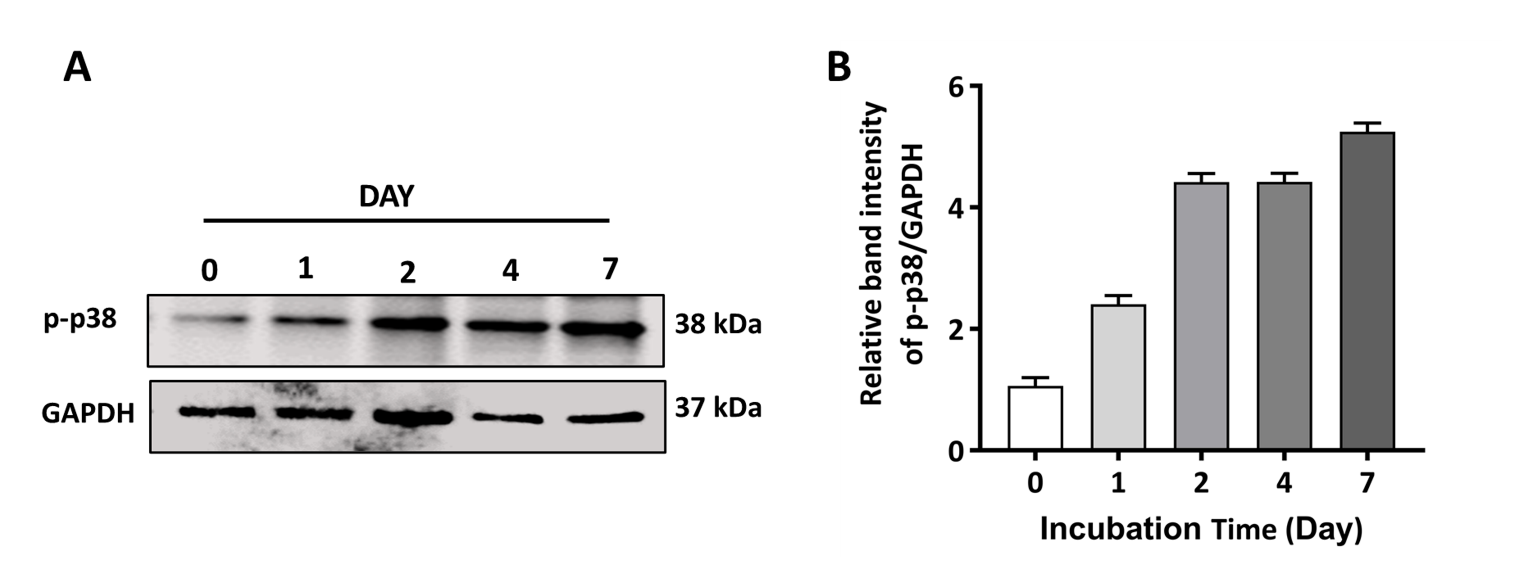** |
| --- |
| **Fig. S5** (A) hBMSCs were cultivated for 0,1, 2, 4, and 7 d after NBP treatment at 4 min and the protein expression of p-p38 was analyzed via western blotting. Without mineral supplementation, the NBP treatment significantly altered the levels of phosphorylated p-38 in the cells (B) The quantification of band intensities is represented in the graphs. The findings showed that p-p38 elevated protein levels in the 4-minute treated group according to the time-dependent manner. GAPDH was used for the normalization. The mean and standard deviation values are used to represent the experimental results. The statistical significance of the data points was assessed using the student's t-test, and significant differences were indicated by * p < 0.05; ** p < 0.01, and *** p < 0.001 vs the untreated control. |
