# Supplementary material for: Nonthermal biocompatible plasma in stimulating osteogenic differentiation by targeting p38/ FOXO1 and PI3K/AKT pathways in hBMSCs
Source: J Biol Eng. 2024 May 28;18:35. doi: 10.1186/s13036-024-00419-2 (PMC11134625; doi:10.1186/s13036-024-00419-2)
Supplement: Supplementary file 2 — Supplementary Material 2. [file 13036_2024_419_MOESM2_ESM.docx]

**Supplemental Table information**

Supplemental Table 1 lists the sequences of the specific primers used to determine the expression levels of the osteogenic target genes, including osteocalcin (OCN), osterix (OSX), alkaline phosphatase (ALP), runt-related transcription factor-2 (Runx2), and collagen type 1 (COL1A1). All the primers were purchased from Life Technologies Corporation Thermo Fisher (USA).

**Table 1: Primer sequences for** **qRT-PCR**

| **Gene** | **Forward** | **Reverse** |
| --- | --- | --- |
| OCN | CACTCCTCGCCCTATTGGC | CCCTCCTGCTTGGACACAAAG |
| COL1A1 | GAGGGCCAAGACGAAGACATC | CAGATCACGTCATCGCACAAC |
| OSX | AGTGACCTTTCAGCCTCCAA | GGGAAAAGGGAGGGTAATCA |
| RUNX-2 | TGGTTACTGTCATGGCGGGTA | TCTCAGATCGTTGAACCTTGCTA |
| ALP | ATGGGATGGGTGTCTCCACA | CCACGAAGGGGAACTTGTC |
| hmTOR | GCA GAT TTG CCA ACT ATC TTC GG | CAG CGG TAA AAG TGT CCC CTG |
| PIK3CA | AGCCACACACTACATCAGTGGCT | ACAGGTCAATGGCTGCATCAT |
| PIK3R1 | TGTCCGGGAGAGCAGTAAACA | CGCCGTCCACCACTACAGA |
| PIK3R2 | AGCTGGACACACGGCTCCT | TGACAATCTGGTCCTGCTGGT |
| GAPDH | ATGGGGAAGGTGAAGGTCG | GGGGTCATTGATGGCAACAATA |
